# The tear film characteristics of spontaneous subconjunctival hemorrhage patients detected by Schirmer test I and tear interferometry

**Published:** 2012-07-18

**Authors:** Wei Liu, Huilin Li, Jun Qiao, Tian Tian, Lin An, Xiaoli Xing, Aihua Liu, Jian Ji

**Affiliations:** 1 Tianjin Medical University Eye Center, Tianjin, China; 2 Tianjin Medical University, Tianjin, China; 3 Eye Department of Changzhi Medical Colledge, Shanxi Province, China; 4 Eye Department of Jingchuan Peoples’ Hospital, Gansu Province, China

## Abstract

**Purpose:**

To evaluate the tear film characteristics of spontaneous subconjunctival hemorrhage patients by Schirmer test I and tear interferometry.

**Methods:**

Forty-six spontaneous subconjunctival hemorrhage patients and 46 controls were enrolled in the study. Schirmer test I and tear interferometry were performed in all 92 subjects. The results obtained were compared between the two groups.

**Results:**

The Schirmer test I value of the spontaneous subconjunctival hemorrhage patients was 6.93 (4.72) mm, and that of the controls was 14.70 (3.70) mm. A statistical difference was found between the two groups (independent samples *t* test, *t*=-8.79, p<0.001). The mean rank of the tear interferometry patterns of the spontaneous subconjunctival hemorrhage patients was 50.07, and that of the controls was 42.93. No statistical difference was found between the two groups (Mann–Whitney U test, *Z*=-1.85, p=0.064).

**Conclusions:**

For the spontaneous subconjunctival hemorrhage patients, the Schirmer test I value was lower than that of the controls, whereas the tear interferometry patterns were comparable to that of the controls.

## Introduction

Subconjunctival hemorrhage (SCH) is a common eye condition, and generally, it is a benign disorder with good visual prognosis. SCH is often characterized by the acute appearance of an area of bleeding under the conjunctiva. Several previous studies have shown the association of SCH with hypertension, diabetes mellitus, trauma, acute hemorrhagic conjunctivitis, anticoagulant therapy, conjunctivochalasis, and Val34Leu mutation of factor XIII [1-12]. It is also well known that the major risk factors for SCH are trauma and contact lens-induced injury in younger patients and hypertension in older patients [4]. Although the clinical features of SCH have been considerably well examined, the tear film characteristics of SCH patients remain unclear. Therefore, this study was performed to evaluate the tear quantity of spontaneous SCH (SSCH) patients by Schirmer test I and the tear quality by tear film interferometry.

## Methods

### Patients and controls

This study adhered to the tenets of the Declaration of Helsinki. This study was approved by the ethics committee of Tianjin Medical University Eye Center, Tianjin, China. Written, informed consent was obtained from all patients and controls. The inclusion criteria for the SSCH group were as follows: first occurrence of SSCH and duration of SSCH <3 days at presentation. The exclusion criteria for the SSCH group included a history of previous SCH, trauma, contact lens use, dry eye, and other diseases that could affect the tear film (blepharitis, dacryocystitis, keratoconjunctivitis sicca, etc). Patients who had a history of systemic disease (including hypertension, diabetes mellitus, and hyperlipidemia) or anticoagulant therapy were also excluded. Patients with cataract or refractive error were enrolled as the control group, and the exclusion criteria were the same as those of the SSCH group. Schirmer test I and tear interferometry were performed in all the SSCH patients and controls.

### Schirmer test I

Schirmer’s strip (Jingming Co. Ltd, Tianjin, China) was placed in the lower fornix near the lateral canthus without anesthesia for 5 min with the patient’s eyes closed. Subsequently, the strip was removed, and the length of the wetted area was measured in millimeters. Every care was taken to avoid touching the cornea.

### Tear interferometry

Noncontact interferometry micrographs of the surface of the tear film were obtained using the DR-1 tear film lipid layer interferometry system (Kowa, Tokyo, Japan). Lipid layer interference images were recorded immediately after a complete blink and were evaluated by an experienced technician. The lipid layer was graded according to a previously reported classification system: grade 1, somewhat gray color, uniform distribution; grade 2, somewhat gray color, nonuniform distribution; grade 3, a few colors, nonuniform distribution; grade 4, many colors, nonuniform distribution; and grade 5, corneal surface partially exposed [13].

### Statistical analysis

Data was reported as mean (SD). All analyses were performed with SPSS software version 13.0. Independent samples *t* test was used for the comparison of age and Schirmer test I results between patients and controls. The Mann–Whitney U test was used for comparison of the tear interferometry patterns between patients and controls. A p<0.05 was considered statistically significant.

## Results

### Patients and controls

From July 2008 to October 2008, 46 SSCH patients and 46 controls were enrolled in this study. The age of the SSCH patients was 57.17 (13.32) years, and the age of the controls was 56.13 (13.71) years—there was no statistical difference in age between the two groups (independent samples *t* test, *t*=0.37, p=0.71).

### Schirmer test I

The result of Schirmer test I for the SSCH patients was 6.93 (4.72) mm, and that for the controls was 14.70 (3.70) mm. A statistically significant difference was found between the two groups (independent samples *t* test, *t*=-8.79, p<0.001).

### Tear interferometry

The mean rank of the tear interferometry patterns of the SSCH patients was 50.07, and that of the controls was 42.93. There was no statistically significant difference between the two groups (Mann–Whitney U test, *Z*=-1.85, p=0.064).

## Discussion

Although SCH is a relatively common eye condition, few studies have addressed the tear film characteristics of SCH patients. However, as already known, most of the risk factors of SCH (such as trauma, contact lens use, diabetes mellitus, etc) affect the stability of the tear film. Therefore, in this study, only the SSCH patients were enrolled, and for the first time, the tear quality and quantity of SSCH patients were assessed.

Since its introduction by Schirmer in 1903, the Schirmer test has been widely used for the assessment of the adequacy of tear production. Schirmer test without anesthesia or Schirmer test I is a well standardized test that measures basal tear secretion with the conjunctival–lacrimal trigeminal reflex [14]. Although the Schirmer test has several drawbacks, it is still considered a useful method to assess tear production [14]. In our study, we found that the Schirmer test values of the SSCH patients were lower than those of the controls, indicating tear production deficiency in SSCH patients. At present, it is unclear why the SSCH eye has aqueous production deficiency, but our hypothesis is as follows. Eyes with aqueous production deficiency present with conjunctival squamous metaplasia and vasculitis of conjunctival blood vessels [15,16], which facilitate the appearance of SCH.

Interferometry is a well known method for the clinical assessment of the integrity of the tear film and the thickness of the lipid layer by observing interference patterns generated by light reflected from the surface of the lipid layer and from the interface between that layer and the aqueous layer of the tear film [17-20]. The commercially available DR-1 interferometer and the classification system of interference patterns used in our study was developed by Yokoi et al. [13]. In this apparatus, the specular reflection from the tear surface is captured using a video camera, fed into a TV monitor, and recorded using a digital video recorder.

Several studies have been performed to evaluate the tear film, especially that of dry eye, by non-invasive interferometry. Using DR-1, Yokoi et al. [13] concluded that tear lipid layer interference patterns were highly correlated with dry eye severity. They found that in the cases of more severe aqueous-deficient dry eyes, the grades of interference patterns were higher, indicating a thicker lipid layer. However, in our study, although the Schirmer test I values of SSCH patients were lower than the controls, the interference patterns were comparable to those of the controls. This unexpected result might be explained by the irregularity of the ocular surface induced by SCH. SCH elevate the conjunctiva and disturb the ocular surface, subsequently affecting the lipid distribution on the cornea, leading to comparable interference patterns.

We acknowledge the following limitations of this study. First, patients with conjunctivochalasis were not excluded from the study, which is a major risk factor for SCH and affects the stability of the tear film. Second, the correlation between the tear film characteristics and the extent of the SCH was not studied. Third, the change in the tear film characteristics was not monitored during the hemorrhage absorption process.

In conclusion, the Schirmer test I values of the SSCH patients were lower than those of the controls, whereas the tear interferometry patterns were comparable to those of the controls.
